# Management of pediatric blunt abdominal trauma in a Dutch level one trauma center

**DOI:** 10.1007/s00068-020-01313-4

**Published:** 2020-02-11

**Authors:** Roy Spijkerman, Lauren C. M. Bulthuis, Lillian Hesselink, Thomas M. P. Nijdam, Luke P. H. Leenen, Ivar G. J. M. de Bruin

**Affiliations:** Department of Trauma Surgery, University Medical Center Utrecht, Heidelberglaan 100, 3584 CX Utrecht, The Netherlands

**Keywords:** Abdominal injuries, Pediatric emergency medicine, Nonpenetrating wounds, Trauma centers

## Abstract

**Purpose:**

Most children with intra-abdominal injuries can be managed non-operatively. However, in Europe, there are many different healthcare systems for the treatment of pediatric trauma patients. Therefore, the aim of this study was to describe the management strategies and outcomes of all pediatric patients with blunt intra-abdominal injuries in our unique dedicated pediatric trauma center with a pediatric trauma surgeon.

**Methods:**

We performed a retrospective, single-center, cohort study to investigate the management of pediatric patients with blunt abdominal trauma. From the National Trauma Registration database, we retrospectively identified pediatric (≤ 18 years) patients with blunt abdominal injuries admitted to the UMCU from January 2012 till January 2018.

**Results:**

A total of 121 pediatric patients were included in the study. The median [interquartile range (IQR)] age of patients was 12 (8–16) years, and the median ISS was 16 (9–25). High-grade liver injuries were found in 12 patients. Three patients had a pancreas injury grade V. Furthermore, 2 (1.6%) patients had urethra injuries and 10 (8.2%) hollow viscus injuries were found. Eighteen (14.9%) patients required a laparotomy and 4 (3.3%) patients underwent angiographic embolization. In 6 (5.0%) patients, complications were found and in 4 (3.3%) children intervention was needed for their complication. No mortality was seen in patients treated non-operatively. One patient died in the operative management group.

**Conclusions:**

In conclusion, it is safe to treat most children with blunt abdominal injuries non-operatively if monitoring is adequate. These decisions should be made by the clinicians operating on these children, who should be an integral part of the entire group of treating physicians. Surgical interventions are only needed in case of hemodynamic instability or specific injuries such as bowel perforation.

**Electronic supplementary material:**

The online version of this article (10.1007/s00068-020-01313-4) contains supplementary material, which is available to authorized users.

## Introduction

The leading cause of morbidity and mortality in children older than 1 year is trauma [1, 2]. In approximately 25% of pediatric patients with major trauma, abdominal injury is present [3, 4]. More than 90% of injuries in children older than 1 year are the result of a blunt traumatic mechanism, with the spleen being the most commonly injured organ [1, 2].

A better understanding of the natural history of intra-abdominal injuries now allows for less aggressive treatment options and more selective operative interventions [5]. A laparotomy is only required in case of a perforating hollow viscus injury or in case of solid organ injury and hemodynamic instability despite adequate resuscitation [6]. In other cases, children with solid organ injuries are generally managed non-operatively [2]. If the solid organ injury is severe (AIS grade 4 and 5), angiographic embolization can be considered to avoid splenectomy [7–11]. Current studies found the ‘older age at the time of splenectomy’ as a protective factor for the development of the overwhelming post-splenectomy infectious syndrome (OPSI) [12]. Therefore, splenic preservation is even more important in younger patients. Angio-embolization could be a useful addition in the treatment protocol to achieve this higher rate of splenic preservation [13, 14]. In the adult population, embolization is well accepted as the treatment of choice in some instances of hemorrhage. However, literature describing the experience with arterial embolization for blunt solid organ injuries in children is limited, but more and more trauma centers are embolizing children.[8, 15–17].

Previous studies have characterized subsets of blunt abdominal injuries in children, looking primarily at either hollow viscus injuries or solid organ injuries [18–21]. However, management of these injuries does not depend on single injuries, but rather on the combination of injuries. Studies examining pediatric intra-abdominal injuries as a whole, including both solid organ injury and hollow viscus injury, are limited.

In Europe, there are many different healthcare systems for the treatment of pediatric trauma patients. In level one trauma centers in The Netherlands, the trauma surgeon is always involved in all steps of clinical decision making and is the leading caretaker, responsible for all the trauma-related problems [22]. This situation is very different from some other trauma centers in Europe (or the rest of the world), where there is super-specialization in pediatric trauma healthcare, and there is not a single person taking into account all different problems together. Literature suggests that trauma patients benefit from a dedicated pediatric trauma surgeon [23, 24]. Alongside this integrated trauma care system, Utrecht has the availability of a dedicated pediatric hospital (Wilhelmina children’s hospital) within the University Medical Center Utrecht. The Wilhelmina children’s hospital has a dedicated pediatric high-end ICU and dedicated pediatric healthcare staff.

Therefore, the aim of this study was to describe the management strategies and outcomes of all pediatric patients with blunt intra-abdominal injuries in our unique dedicated pediatric trauma center with a pediatric trauma surgeon.

## Materials and methods

### Study design

A retrospective, observational study was performed to investigate the management of pediatric blunt abdominal trauma in the University Medical Center Utrecht (UMCU). For this analysis, a waiver was provided by the institutional medical ethics committee under protocol number 18–789. In addition, in line with the academic hospital policy, an opt-out procedure is in place for the use of patient data for research purposes. The process and storage of data are following privacy and ethics regulations.

### Patients

The UMCU is a Joint Commission International (JCI) accredited tertiary care facility with 1000 beds and a regional referral center of the region ‘Midden-Nederland’ that inhabits 2.2 million people. The UMCU is connected with several referral hospitals for the transfer of trauma patients. A part of the UMCU is a separate dedicated pediatric hospital, the Wilhelmina Children’s hospital (Dutch: Wilhelmina Kinder Ziekenhuis (WKZ)). Our hospital complies with all requirements as defined by the American College of Surgeons’ Committee on Trauma (ACS-COT) for a Level 1 Trauma Center. All children admitted at the emergency department will be transferred to the dedicated pediatric hospital after initial treatment if necessary. Children were all treated according to the American Pediatric Surgical Association (APSA) and the American Association for the Surgery of Trauma (AAST) guidelines. From the National Trauma Registration database, we retrospectively identified patients presented to the UMCU. This national trauma registration contains data from patients who were treated in an emergency department (ED) within 48 h after the accident and subsequently admitted to a hospital for treatment or patients who died in the ED. Included were all patients < 18 years admitted to the UMCU diagnosed with a blunt abdominal injury during the period starting from January 1, 2012, to December 31, 2017. Patients were excluded when they deceased on arrival or in the emergency department.

All patient charts and follow-up files were reviewed, and patient characteristics, trauma characteristics, diagnostic workup, treatment, and outcome were documented. Patient and trauma characteristics included age in years, gender, transfer from another hospital, mechanism of injury, systolic blood pressure (SBP) in millimeter of mercury, pulse rate (PR) in beats per minute, respiration rate (RR) in number of breaths per minute, Glasgow Coma Score (GCS), serum hemoglobin (Hb) in millimole per liter, pH, lactate (mmol/l). Injuries were graded based on the American Association for Surgery of Trauma organ injury grading scales. All injuries were scored according to the Abbreviated Injury Scale (AIS) by an [25] authorized registrar by analyzing the Computed tomography (CT)–scan, diagnostic abdominal ultrasound (US), or after abdominal exploration [26]. The injury severity score (ISS) was calculated per patient after dismissal. The ISS was calculated with version’98 until January 13, 2015; afterwards, the ISS’08 was used. The research team scored a patient as hemodynamic unstable if hemodynamic instability was noted in the electronic patient registry by the trauma surgeon on duty. The most used parameters to assess hemodynamic instability are both SBP < 80–100 and HR > 100–120 [6]. In the case of hemodynamic instability, the mass transfusion protocol was always started using the ratio one package red blood cells on one package fresh frozen plasma on one package platelets. Concomitant severe head and severe thorax injuries were scored when AIS ≥ 3. Extremity injuries were scored as concomitant injury if they were graded AIS ≥ 2.

Patients were categorized by the type of treatment they initially received. The first group consisted of patients treated by non-operative treatment (NOM). They underwent observational treatment alone or were treated by observational treatment with the addition of angioembolization (AE). Following institutional treatment protocol, AE was only used in patients ≥ 16 years. Patients who received operative management (OM) were included in the second group. OM was defined as an initial treatment with an emergency laparotomy. Operative reports were reviewed for indications for operative interventions. The outcome measurements of this study included post-operative abdominal complications, length of hospital stay (LOS), length of intensive care unit (ICU)-stay, (re-)intervention for abdominal complications and mortality. Complications were recorded by the treating physician in the complication registry using the International Classification of Diseases, Tenth Revision (ICD-10) [27].

### Statistical analysis

All data were analyzed with SPSS version 25.0.0.2 (IBM Corporation, NY, United States). The distribution of continuous variables was assessed with the use of the Kolmogorov–Smirnov test. Results were presented as median with interquartile range (IQR), because the data were not normally distributed. A comparison of baseline and outcome characteristics between groups was performed with a Chi-square/Fisher’s exact test or a Mann–Whitney U test, as indicated. Statistical significance was defined as a *P* value < 0.05.

## Results

**Fig. 1:**
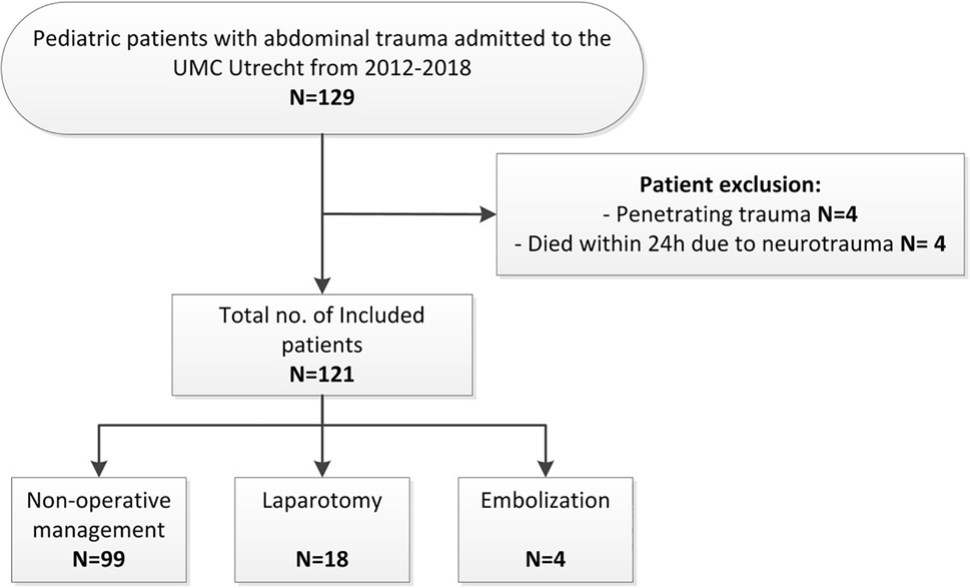
Flowchart

A total of 129 children with abdominal injuries were identified. Of these patients, four patients were excluded due to penetrating trauma, and four because of death within 24 h due to traumatic brain injury. Therefore, a total of 121 patients were included in this study (Fig. 1). Baseline characteristics are shown in Table 1. The study group consisted of 83 (68%) males and 38 (31%) females, with a median age of 12 (8–16) and a median ISS of 16 (9–25). On admission, the study group had a median SBR of 120 (108–130), a PR of 92 (80–110), and a GCS of 15 (14–15). A total of 47 (39%) patients were transferred from a different hospital to the UMC Utrecht. The mechanisms of injury below the age of 12 fell from a height in 44% of the patients, bicycle accident in 30%, a car accident in 15%, pedestrian hit by a moving vehicle in 5% and other in 7%. In children older than 12 years, the main mechanisms of injury were motorcycle accident (34%), bicycle accident (23%), car accident (17%), fall from height (11%), and a pedestrian hit by a moving vehicle (4%). Other mechanisms of injury included two sports injuries, one suicide attempt, one assault by human, three assaults by animals, one running into a fence, and one agriculture vehicle collision. In 90 children (55%), there were severe concomitant injuries. Severe traumatic brain injuries were found in 19 (16%) patients, severe thorax injuries in 38 (32%) patients, and severe injury to extremities in 33 (27%) patients.

**Table 1:** Baseline characteristics of included patients

	Total (*N* = 121)	Group I:NOM (*N* = 103)	Group II: OM (*N* = 18)	*P* value
Age at trauma, years	12 (8–16)	12 (8–16)	16 (11–18)	0.254
Gender (M/F)	83/38	71/32	12/6	0.709
Injury Severity Score	16 (9–25)	16 (9–23)	28 (18–35)	0.001*
**Mechanism of injury**				
*Fall ≤ 3 m*	27 (22%)	25 (25%)	2 (11%)	
*Fall > 3 m*	5 (4%)	5 (5%)	0 (0%)	
*Bicycle accident*	30 (25%)	27 (27%)	3 (18%)	
*Motorcycle accident*	24 (20%)	18 (17%)	6 (33%)	
*Car accident*	19 (16%)	14 (14%)	5 (27%)	
*Pedestrian in MVA*	6 (5%)	6 (6%)	0 (0%)	
*Other* ^a^	10 (8%)	8 (8%)	2 (11%)	
**No. patients with** **Concomitant injuries**				
*Severe head injury*	19 (15%)	17 (16%)	2 (11%)	0.476
*Severe thorax injury*	38 (31%)	31 (30%)	7 (39%)	0.349
*Extremity injury*	33 (27%)	26 (25%)	7 (39%)	0.423
Transfer from other hospital	47	45	2	0.015*
Glasgow Coma Score	15 (14–15)	15 (14–15)	14 (3–15)	0.017*
Pulse rate	92 (80–110)	90 (80–105)	110 (89–120)	0.031*
Systolic blood pressure	120 (108–130)	120 (110–130)	119 (94–126)	0.076
Respiratory rate	18 (15–22)	18 (15–21)	21 (15–25)	0.266
Serum Haemoglobin	7.7 (7.0–8.3)	7.8 (7.1–8.4)	7.2 (5.5–8.3)	0.017*
pH	7.4 (7.3–7.4)	7.4 (7.3–7.4)	7.2 (7.1–7.4)	0.004*
Lactate	2.3 (1.2–3.6)	2.2 (1.1–3.3)	3.7 (2.0–7.9)	0.053
Base excess	-2.0 ((-4.0)-0.0)	-2.0 ((-3.5)-0.0)	-7.0 ((-12.7)-(-0.7))	0.002*

All variables are in total amount (percentage) or median (IQR)

*MVA* motor vehicle accident, *NOM* Non-operative management, *OM* operative management

^a^Other: Sports (*n* = 2), suicide attempt (*n* = 1), assault by other (*n* = 1), assault by animal (*n* = 3), running into fence (*n* = 2), agriculture vehicle (*n* = 1)

* P*-values marked with asterisk (*) are significant

Initially, a total of 22 out of 121 patients (18%) required immediate intervention, of whom 18 patients underwent laparotomy, and 4 patients underwent AE. Two patients with an active blush from their grade 4 liver injury and 2 patients with an active blush from their grade 4 splenic injury were treated by AE. Patients in the laparotomy group showed the highest pulse rate of 110 (89–120) and respiratory rate 21 (15–25). Furthermore, ISS was higher in patients undergoing laparotomy. Of the patients treated by NOM, 17 patients (17%) had severe head injuries, 31 patients (30%) had severe thorax injuries, and 26 patients (25%) had injuries to the extremities. In the laparotomy group, 2 (11%) had severe head injuries, 7 (39%) had severe thorax injuries, and 7 (39%) had injuries to the extremities. There were no significant differences found in concomitant injuries between the study groups.

Of the 121 children diagnosed with abdominal injuries, a total of 116 (96%) were scanned by CT-scan. In 30 of these patients (25%), CT-scan was preceded by focused assessment with sonography in trauma (FAST). The radiologist on-duty performed FAST in all cases. Five children did not receive a CT-scan. Of these patients, diagnostic US was used to diagnose abdominal injuries in three patients. Because only minor injuries were suspected, no CT-scan was obtained. The other two patients were not scanned because of hemodynamic instability. In these patients, FAST followed by the operative intervention was considered the diagnostic work-up.

An overview of the abdominal injuries is shown in Table 2. A laparotomy was performed in 18 patients. In nine out of 18 patients, the reason for laparotomy was hemodynamic instability due to solid organ injuries. Solid-organ injuries that resulted in hemodynamic instability included five splenic injuries and four liver injuries. A splenectomy was performed in four patients, and packing was performed in one patient with splenic bleeding and all patients with liver injuries. In the nine other patients, the reason for surgical intervention was the specific type of injury. In three of these patients, a fully transected pancreas with duct injury was found, which was managed by resection of the tail in one patient and pancreas repair in the other two patients. In the six other patients, the indication for surgery was bowel perforation.

**Table 2:** Overview of the abdominal injuries found after initial diagnostic modality

	Total (*N* = 121)	Group I: NOM (*N* = 103)		Group II: OM (*N* = 18)	
**Abdominal solid organ injuries**^a^					
Kidney					
*Grade I/II*	20 (17%)	18 (18%)			2 (11%)
* Grade III*	2 (2%)	1 (1%)			1 (6%)
*Grade IV*	1 (1%)	1 (1%)			0 (0%)
*Grade V*	0 (0%)	0 (0%)			0 (0%)
Liver					
*Grade I/II*	16 (13%)	15 (15%)			1 (6%)
*Grade III*	15 (12%)	13 (13%)			2 (11%)
*Grade IV*	12(10%)	10 (10%)			2 (11%)
*Grade V*	2 (2%)	0 (0%)			2 (11%)
Pancreas					
*Grade I/II*	1 (1%)	1 (1%)			0 (0%)
*Grade III*	2 (2%)	2 (2%)			0 (0%)
*Grade IV*	0 (0%)	0 (0%)			0 (0%)
*Grade V*	3 (3%)	0 (0%)			3 (17%)
Spleen					
*Grade I/II*	23 (19%)	18 (18%)			5 (28%)
*Grade III*	19 (16%)	17 (17%)			2 (11%)
*Grade IV*	15 (12%)	14 (14%)			1 (6%)
*Grade V*	2 (2%)	0 (0%)			2 (11%)
**Abdominal hollow viscus injuries**^a^					
Rectum					
*Grade III*	1 (1%)	0 (0%)	1 (6%)		
Colon					
*Grade II*	2 (3%)	0 (0%)	2 (11%)		
*Grade III*	1 (1%)	0 (0%)	1 (6%)		
Duodenum					
*Grade II*	4 (3%)	1 (1%)	3 (17%)		
Small bowel					
*Grade II*	2 (%)	2 (1%)	0 (0%)		
*Grade III*	1 (%)	0 (0%)	1 (6%)		
*Grade IV*	1 (%)	0 (0%)	1 (6%)		
**Diaphragm**	2 (2%)	1 (1%)	1 (6%)		
**Vascular**	2 (2%)	1 (1%)	1 (6%)		
**Urogenital**	3 (3%)	0 (0%)	3 (17%)		

All variables are in total amount(percentage)

*NOM* non-operative management, *OM* operative management

^a^Brief description of organ injury scale (supplement 1)

In total, five hollow viscus injuries required surgical intervention. In one patient, grade 3 colon injury required suturing, in two patients small bowel resection was needed and in one patient a rectum injury needed resection. There were three patients with duodenal injuries; in one of these patients, surgical closure was indicated. Furthermore, one urethra injury was found for which a suprapubic catheter was placed surgically. In addition, one hemipelvectomy was performed in a patient with irreparable nerve and vascular damage. Finally, one patient, primarily treated non-operatively, received surgery after increased abdominal pain and inexplicable free abdominal air, a rupture of the diaphragm was found and primarily sutured.

ICU and hospital length of stay were significantly longer in patients who underwent a laparotomy compared to other patients with abdominal injuries (*P* = 0.003 and *P* < 0.001, respectively). Complications occurred in 6 out of 121 patients. Three patients in the NOM group developed complications (3%) and three patients in the OM group (17%). All complications are listed in Table 3. The most prevalent complication in our study was a rebleed, which complicated the clinical course of two patients (2%) in the NOM group. These patients became hemodynamically unstable during hospitalization. The first patient was transferred from a different hospital where computed tomography angiography (CTA) showed a subcapsular liver hematoma with no active blush.

**Table 3:** Outcome measurements

	Total (*N* = 121)	Group I: NOM (*N* = 103)	Group II: OM (*N* = 18)	*P* value
No. of patients with complications	7 (6%)	4 (4%)	3 (17%)	0.032*
**Abdominal complications**				
*Rebleed*	2 (2%)	2 (2%)	0 (0%)	
* Infected hematoma*	2 (2%)	1 (1%)	1 (6%)	
* Bile leak*	2 (2%)	0 (0%)	2 (11%)	
* Abscess*	1 (1%)	0 (0%)	1 (6%)	
* Empyema*	1 (1%)	0 (0%)	1 (6%)	
* Urinoma*	1 (1%)	1 (1%)	0 (0%)	
* Missed diaphragm injury*	1 (1%)	1 (1%)	0 (0%)	
No. of patients requiring intervention for abdominal complications	5 (4%)	4 (4%)	1 (6%)	0.560
**Non-abdominal complications**				
* Pneumonia*	7 (6%)	5 (5%)	2 (11%)	
*Fever unspecified*	5 (4%)	5 (5%)	0 (0%)	
* Allergic rash*	3 (2%)	2 (2%)	1 (6%)	
*Delirium*	5 (4%)	3 (3%)	2 (11%)	
*Pleural effusion*	1 (1%)	1 (1%)	0 (0%)	
* Retention of urine*	1 (1%)	1 (1%)	0 (0%)	
* Phlebitis*	2 (2%)	2 (2%)	0 (0%)	
*Compartment syndrome*	1 (1%)	1 (1%)	0 (0%)	
*Pressure ulcers*	1 (1%)	1 (1%)	0 (0%)	
* Arterial embolism*	1 (1%)	0 (0%)	1 (6%)	
No. of patients requiring intervention for non-abdominal complications	2 (2%)	1 (1%)	1 (6%)	0.276
Length of hospital stay in days	6 (4–11)	4 (3–7)	15 (7–34)	< 0.001*
Length of ICU stay in days	1 (1–2)	1 (1–2)	2 (1–9)	0.003*
Mortality	1 (1%)	0 (0%)	1 (6%)	0.140

All variables are in total amount (percentage) or median (IQR)

*ICU* intensive care unit, *NOM*  non-operative management, *OM*  operative management, *NA* not applicable

*P*-values marked with asterisk (*) are significant

The patient was admitted to the ICU for observation. Two days after admission, the patient became hemodynamically unstable, and active bleeding was suspected. Embolization of the arteria hepatica sinistra and dextra was performed. After the intervention, the patient was hemodynamically stable, but due to persistent abdominal pain, a laparotomy was performed, which showed a biloma that required drainage. The second patient was admitted to our hospital with a grade four liver injury. Because the patient was hemodynamically stable, no surgical intervention was indicated, and she was admitted to the pediatric ward for observation. Two days after admission, the patient had tachycardia and decreasing hemoglobin levels. Angiography demonstrated three active blushes from the liver, and embolization was performed. Other complications included an infected hematoma in a NOM patient, a bile leak, an abscess and lung empyema in an OM patient, and another bile leak in an OM patient.

All kidney injuries were initially conservatively treated. Only grade four kidney injury developed a urinoma and required a JJ-stent. Intervention for complications was needed in three cases in the NOM group (3%) and one case in the OM group (6%). In the NOM group, the causes were the occurrence of a rebleed in the two patients mentioned above and an infected hematoma in one patient. None of these patients died. One of the 18 laparotomy patients died after several days, due to the propofol infusion syndrome.

## Discussion

In this study, we investigated the incidence and management of intra-abdominal injuries in children in our institution. In our study cohort of children with intra-abdominal injuries, low-grade splenic injuries were most frequently found, followed by low-grade liver injuries. We also found that high-grade liver and spleen injuries were approximately equal in frequency. Initial operative or interventional treatment of kidney injuries was not necessary. High-grade pancreatic injuries occurred in only 2.5% of all patients and required surgical intervention in all cases.

Overall, the rate of surgical interventions (15%) in children with intra-abdominal trauma was in line with other pediatric studies, including hollow viscus injuries as well [28]. However, a decrease in surgical interventions might be possible with the introduction of angioembolization in young children. In this study, all embolization procedures were performed in children > 16 years with high-grade liver or spleen injury. In the adult population, angiographic embolization is a standard treatment used for blunt abdominal injuries, but in pediatric patients, angiographic embolization is rarely used [11, 16, 17, 29]. Skattum et al. demonstrated that the use of splenic angioembolization could increase the use of NOM with splenic preservation from 90 to 98% pediatric patients [30]. In addition, Sweed et al. described four cases of pediatric trauma and demonstrated that angiographic embolization could have value in stabilization and treatment [11]. However, they mentioned that this method requires highly skilled personnel and the availability of adequate equipment [11]. Therefore, it is likely that if all requirements were present, some laparotomies in these children could have been prevented by performing AE. A recent literature review showed improving long term effects of AE in blunt splenic injury [13]. In this review, almost all studies that performed splenic function tests showed a preserved splenic function after AE. In case of an inadequate splenic function, the infection prevention protocol should be applied [14]. However, there is still no single parameter or test available, which can demonstrate a preserved splenic function. The local institutional protocol in the UMC Utrecht did not implement SAE for patients < 16 years yet, because the 24/7 infrastructure in pediatric AE is not yet established. Furthermore, due to recent progression in the field of endoscopic interventions, it is now possible to treat grade 4 and 5 pancreas injuries non-operatively [31–33]. In the inclusion period, there were three patients with grade 5 pancreas injuries. Operative management of these patients could have been avoided if endoscopic management was successfully done. However, at the time of patient inclusion, this was not current practice yet.

Complications were rare in both operatively managed, and non-operatively managed patients. Especially in the NOM group, the complication rate was considerably lower (5%) than described in the literature [25, 34–37]. Re-intervention, due to these complications, such as re-bleed or bile leak, was required in only a few cases. Two patients managed by NOM developed hemodynamic instability during admission, but due to strict hemodynamic monitoring and immediate intervention, mortality and additional morbidity could be avoided. The availability of modern facilities nowadays enables immediate intervention when needed, which allows for a more liberal selection of NOM patients and better outcomes of NOM.

Furthermore, when a multidisciplinary team provides care, there must be a leading practitioner to take responsibility in management decisions, preferably the clinician who operates the child. In our hospital, the trauma surgeon was always involved in clinical decision making. This clinician thoroughly considered NOM and OM for every specific case, taking into account patient and injury characteristics and took responsibility for adequate monitoring. Therefore, reasons for a low complication rate found in this study could be that there was always one trauma surgeon taking the final decisions, that NOM and OM were thoroughly considered for every single case and that NOM patients were closely monitored.

CT-scan is considered the most accurate modality to diagnose and grade abdominal injuries, with negative predictive values greater than 99% [38–40]. In this study, a CT-scan was performed in 96% of all patients. Because of this, we were able to give an accurate overview of all abdominal injuries in our pediatric trauma population over the last 5 years. The strength of our study is that we used data from a large patient group, treated according to recent APSA and AAST guidelines in a dedicated pediatric level one trauma center linked to a dedicated pediatric hospital [41, 42]. Furthermore, this study is unique in describing intra-abdominal trauma as a whole, including both solid organ injury and hollow viscus injury.

Our study also has certain limitations; the study population represents the practice at only one institution, and thus it may not reflect practice at all hospitals. Furthermore, this study has a retrospective design, potentially causing a selection or information bias. However, due to a strictly maintained trauma data registry, we were able to identify a large number of patients, and we had unlimited excess to all patient charts and laboratory results. Hence, the chance of under-registration of findings was considered to be very low.

In conclusion, it is safe to treat most children with blunt abdominal injuries non-operatively if monitoring is adequate, and the institute has expertise with pediatric trauma patients. These decisions should be made by experienced clinicians, treating these children, who should be an integral part of the entire group of treating physicians. Surgical interventions are only needed in case of hemodynamic instability or specific injuries such as bowel perforation.

## Electronic supplementary material

Below is the link to the electronic supplementary material.

Supplementary file1 (DOCX 21 kb)
