# Supplementary material for: Management of pediatric blunt abdominal trauma in a Dutch level one trauma center
Source: Eur J Trauma Emerg Surg. 2020 Feb 11;47(5):1543–51. doi: 10.1007/s00068-020-01313-4 (PMC8476366; doi:10.1007/s00068-020-01313-4)
Supplement: Supplementary file 1 — Supplementary file1 (DOCX 21 kb) [file 68_2020_1313_MOESM1_ESM.docx]

**Supplement 1 (AAST organ injury score**[26]**) 🡪**

| Grade | Type of kidney injury | Description |
| --- | --- | --- |
| I | Contusion | Microscopic or gross hematuria, urologic studies normal |
|  | Hematoma | Subcapsular, nonexpanding, without parenchymal laceration |
| II | Hematoma | Nonexpanding perirenal hematoma limited to renal retroperitoneum |
|  | Laceration | <1.0-cm parenchymal depth of renal cortex without urinary extravasation |
| III | Laceration | <1.0-cm parenchymal depth of renal cortex without collecting system rupture or urinary extravasation |
| IV | Laceration | Parenchymal laceration that extends through renal cortex, medulla, and collecting system |
|  | Vascular | Main renal artery or vein injury with contained hemorrhage |
| V | Laceration | Completely shattered kidney |
|  | Vascular | Avulsion of renal hilum that devascularizes kidney |

| Grade | Type of hepatic injury | Description |
| --- | --- | --- |
| I | Hematoma | Subcapsular, <10% surface area |
|  | Laceration | Capsular tear, <1-cm parenchymal depth |
| II | Hematoma | Subcapsular, 10%-50% surface area; intraparenchymal, <10 cm in diameter |
|  | Laceration | Capsular tear, 1-3–cm parenchymal depth, <10 cm in length |
| III | Hematoma | Subcapsular, >50% surface area of ruptured subcapsular or parenchymal hematoma; intraparenchymal hematoma ≥10 cm or expanding |
|  | Laceration | >3-cm parenchymal depth |
| IV | Laceration | Parenchymal disruption that involves 25%-75% of a hepatic lobe or 1-3 Couinaud segments within a single lobe |
| V | Laceration | Parenchymal disruption that involves >75% of a hepatic lobe or >3 Couinaud segments within a single lobe |
|  | Vascular | Juxtahepatic venous injuries (retrohepatic vena cava or major hepatic veins) |
| VI | Vascular | Hepatic avulsion |

| Grade | Type of splenic injury | Description |
| --- | --- | --- |
| I | Hematoma | Subcapsular, <10% surface area |
|  | Laceration | Capsular tear, <1-cm parenchymal depth |
| II | Hematoma | Subcapsular, 10%-50% surface area; intraparenchymal, <5 cm in diameter |
|  | Laceration | Capsular tear, 1-3–cm parenchymal depth that does not involve a trabecular vessel |
| III | Hematoma | Subcapsular, >50% surface area or expanding; ruptured subcapsular or parenchymal hematoma; intraparenchymal hematoma ≤5 cm or expanding |
|  | Laceration | >3-cm parenchymal depth or involving trabecular vessels |
| IV | Laceration | Laceration that involves segmental or hilar vessels, producing major devascularization (>25% of spleen) |
| V | Laceration | Completely shattered spleen |
|  | Vascular | Hilar vascular injury with devascularized spleen |

| Grade | Type of pancreatic injury | Description |
| --- | --- | --- |
| I | Hematoma | Minor contusion without duct injury |
|  | Laceration | Superficial laceration without duct injury |
| II | Hematoma | Major contusion without duct injury or tissue loss |
|  | Laceration | Major laceration without duct injury or tissue loss |
| III | Laceration | Distal transection or parenchymal injury with duct injury |
| IV | Laceration | Proximal transection or parenchymal injury involving ampulla |
| V | Laceration | Massive disruption of pancreatic head |

| Grade | Type of colon injury | Description |
| --- | --- | --- |
| I | Hematoma | Contusion or hematoma without devascularization |
| II | Laceration | Partial thickness, no perforation |
|  | Laceration | Laceration <50% of circumference |
| III | Laceration | Laceration > 50% of circumference without transection |
| IV | Laceration | Transection of the colon |
| V | Laceration | Transection of the colon with segmental tissue loss |
|  | Vascular | Devascularized segment |

| Grade | Type of duodenum injury | Description |
| --- | --- | --- |
| I | Hematoma | Involving single portion of duodenum |
|  | Laceration | Partial thickness, no perforation |
| II | Hematoma | Involving more than one portion |
|  | Laceration | Disruption <50% of circumference |
| III | Laceration | Disruption 50%-75% of circumference of D2 |
|  |  | Disruption 50%-100% of circumference of D1,D3,D4 |
| IV | Laceration | Disruption >75% of circumference of D2 |
|  |  | Involving ampulla or distal common bile duct |
| V | Laceration | Massive disruption of duodenopancreatic complex |
|  | Vascular | Devascularization of duodenum |

| Grade | Type of small bowel injury | Description |
| --- | --- | --- |
| I | Hematoma | Contusion or hematoma without devascularization |
| II | Laceration | Partial thickness, no perforation |
|  | Laceration | Laceration <50% of circumference |
| III | Laceration | Laceration > 50% of circumference without transection |
| IV | Laceration | Transection of the small bowel |
| V | Laceration | Transection of the small bowel with segmental tissue loss |
|  | Vascular | Devascularized segment |
